# Theta and Alpha Oscillations Reflect Distinct Control and Stabilization Processes Across Working Memory

**DOI:** 10.3390/brainsci16060625

**Published:** 2026-06-11

**Authors:** Adrián Ávila-Garibay, Geisa B. Gallardo-Moreno, Fabiola R. Gómez-Velázquez, Steven Woltering, Andrés A. González-Garrido

**Affiliations:** 1 Instituto de Neurociencias, Universidad de Guadalajara, Guadalajara C.P. 44130, Jalisco, Mexico; adrian.avila5757@alumnos.udg.mx (A.Á.-G.); geisa.gallardo@academicos.udg.mx (G.B.G.-M.); fabiola.gomez@academicos.udg.mx (F.R.G.-V.); 2 Department of Educational Psychology, Texas A&M University, College Station, TX 77843-4225, USA; swolte@tamu.edu

**Keywords:** working memory, Sternberg task, EEG, theta, alpha

## Abstract

**Highlights:**

**What are the main findings?**

- During the retrieval, where the participants responded to the probe, theta power was higher than in earlier working memory stages.
- Upper alpha power was associated with increased probability of correct responses, highlighting the role of specific oscillatory activity in performance.

**What are the implications of the main findings?**

- Theta oscillations may index the coordination of processes involved in task responses, such as accessing stored information and decision-making.
- Upper alpha activity may support performance in demanding cognitive tasks.

**Abstract:**

**Background/Objectives**: The oscillatory dynamics underlying stage-specific processing in working memory (WM) remain incompletely characterized, particularly under varying memory loads. We examined the load-dependent modulation of theta (4–7 Hz), lower alpha (8–10 Hz), and upper alpha (11–13 Hz) absolute power during encoding, maintenance, and retrieval using quantitative EEG in a modified Sternberg task that temporally dissociates these stages. **Methods**: Forty-five healthy young adults performed trials with memory sets of three, five, or six uppercase consonants, followed by a lowercase probe. EEG data were analyzed using cluster-based permutation testing, and brain–behavior relationships were assessed using regression models. **Results**: Fronto-central theta power increased with memory load and was significantly higher during retrieval than during encoding or maintenance. Greater theta power during retrieval predicted faster reaction times in the three-letter condition. Alpha oscillations showed robust stage effects. Lower alpha power was higher during maintenance than retrieval across loads and exhibited a load effect during maintenance (three > six letters) in occipital regions. Upper alpha power was consistently maximal during maintenance across all loads, involving bilateral fronto-central, parietal, and occipital regions. Critically, under moderate load (five letters), higher upper alpha power predicted a greater probability of correct responses across task stages. **Conclusions**: These findings demonstrate a functional dissociation between oscillatory bands across temporally separated WM stages: theta activity was retrieval-dominant and associated with response speed, whereas alpha, particularly upper alpha, was maintenance-dominant and supported accuracy under increased mnemonic demand.

## 1. Introduction

Working memory (WM) is among the most extensively studied cognitive constructs. It refers to the cognitive system responsible for the temporary storage and manipulation of information necessary for complex tasks such as reasoning, problem-solving, and language comprehension [1].

WM processing is commonly divided into three broad stages: encoding, maintenance/manipulation, and retrieval [2]. These stages depend on coordinated activity across distributed neural networks, particularly involving the prefrontal cortex, which plays a central role in attentional regulation, executive control, and decision-making [3,4]. Because WM capacity is limited, cognitive load strongly influences performance across all processing stages [5]. Cognitive load theory proposes that exceeding the processing limits of WM results in cognitive overload and reduced task efficiency [6]. This relationship between load and WM performance has been well documented; for example, higher WM load has been associated with impairments in reasoning and problem-solving performance [7]. However, despite general agreement about the impact of cognitive load, the precise effects of increasing load on attentional control and distractor processing remain debated. Some studies suggest that higher WM load increases distractor interference [8], whereas others propose that increasing perceptual or WM load reduces distractor processing as cognitive demands rise [9]. These discrepancies may depend on the nature of the task and the type of cognitive load involved [10].

Extending these considerations to the neural level, neuroimaging studies have identified several neural substrates associated with WM, including dorsolateral prefrontal and posterior sensory cortices [1,11]. Nevertheless, many of these approaches provide limited temporal resolution and therefore cannot fully characterize the fast neural dynamics underlying the successive stages of WM processing. To address this issue, electrophysiological methods, particularly quantitative EEG (qEEG), offer an important advantage because they can capture rapid oscillatory changes associated with encoding, maintenance, and retrieval processes [12]. Among EEG frequency bands, theta and alpha oscillations have been consistently linked to attention, long-term memory, and WM performance [13,14,15]. However, despite extensive research, it remains unclear how these oscillations are differentially modulated across WM stages, memory loads, and cortical regions.

Current evidence suggests that theta oscillations play distinct roles in executive and attentional processes during WM. Frontal theta power increases during stimulus presentation, reflecting involvement in early encoding and attentional allocation processes regardless of sensory modality [16]. In occipital regions, theta increases immediately after stimulus presentation, while frontal theta activity is enhanced during n-back tasks [17]. In right frontal areas, greater theta activity occurs during encoding compared to retrieval in visuospatial virtual reality tasks [18]. Theta oscillations are also sensitive to memory load, as shown by increased frontal and prefrontal theta power with higher task demands [19,20]. Notably, sustained theta activity is present throughout encoding, maintenance, and retrieval, indicating broad WM contributions [16]. However, inconsistencies persist. For example, intracranial recordings from epilepsy patients showed increased theta activity during maintenance in the hippocampus and dorsal anterior cingulate cortex, but decreased theta power in the dorsolateral prefrontal cortex as memory load increased [21]. These findings imply that theta oscillations support distinct cognitive operations across different processing stages and cortical regions.

Alpha oscillations are also closely associated with distinct processing, with evidence indicating that processing stages are associated with the inhibition of distracting information and modulating attentional control. Several studies show that alpha power generally decreases with increasing perceptual processing demands, especially in frontal regions during maintenance and retrieval [17], interpreted as increased cortical engagement. Reductions in parietal alpha power are linked to heightened arousal and resource allocation [20,22]. However, increases in frontal alpha during maintenance suggest active inhibition of irrelevant information [23]. Additionally, alpha increases during visuospatial manipulation indicate a role in the handling of complex cognitive resources [24]. Thus, alpha oscillations are implicated both in suppressing distraction via inhibition (frontal regions) and directing attention or reallocating resources (parietal regions). However, the direction and regional distribution of alpha modulation still vary, making it challenging to definitively assign inhibitory control or attentional engagement as its primary function.

A further challenge relates to the functional specificity within the alpha band. Studies suggest that lower alpha (approximately 8–10 Hz) is primarily involved in attentional gating and general task engagement, helping to filter out irrelevant information, while upper alpha (approximately 10–13 Hz) appears to be linked to semantic processing, memory load, and top-down executive control [12,14,25,26,27]. In WM tasks, upper alpha power often increases with memory load, signaling support for active maintenance and manipulation, whereas lower alpha may facilitate attentional disengagement from nonessential sensory input. These findings indicate that lower and upper alpha serve partially distinct functions during WM, with the former oriented towards attentional filtering and the latter towards memory processing and cognitive control. However, few studies examine these sub-bands separately across WM stages, limiting clarity about their unique contributions.

Despite these advances, a major limitation of the existing literature is that many WM paradigms, particularly the n-back task, involve simultaneous encoding, updating, maintenance, and retrieval operations [28,29]. Because these processes overlap temporally and recruit widespread neural networks, it is difficult to determine which oscillatory changes correspond primarily to a specific processing stage. Moreover, although theta and alpha oscillations have repeatedly been associated with WM load, previous findings remain inconsistent regarding how these bands vary across encoding, maintenance, and retrieval, as well as across frontal and posterior cortical regions. Addressing this gap requires the simultaneous examination of the processing stage, memory load, and regional oscillatory activity using a task design that clearly separates WM stages, an approach that few studies have taken.

To overcome these challenges, the present study was designed to address the unresolved issues identified above. Specifically, we used a modified Sternberg item-recognition task that allows the sequential dissociation of encoding, maintenance, and retrieval processes, combined with qEEG measures of absolute theta and alpha power. In addition, lower and upper alpha sub-bands were analyzed separately to determine whether they exhibit differential modulation across WM stages and memory load conditions. By examining oscillatory activity across fronto-central and parieto-occipital regions under different memory loads, this study aims to characterize the temporal, regional, and load-dependent dynamics of theta and alpha oscillations during WM processing.

Based on previous findings and the aforementioned rationale, we hypothesized that theta oscillations would show load-dependent increases and stage-specific modulation reflecting executive engagement and attentional allocation. We also expected alpha oscillations to exhibit stage-dependent changes, particularly during maintenance, consistent with their proposed inhibitory and attentional-control functions. Furthermore, we anticipated that lower and upper alpha bands would display partially distinct modulation patterns across WM stages and cortical regions. By clarifying the temporal and regional specificity of theta and alpha dynamics, the present study seeks to refine current neurophysiological models of WM and improve the interpretation of qEEG markers in both experimental and clinical contexts.

## 2. Materials and Methods

### 2.1. Participants

Forty-five healthy, right-handed university individuals (23 female, 22 male) between 20 and 30 years old (*M* = 23, *SD* = 2) with typical estimated IQs (WAIS-IV (Manual Moderno, CDMX, Mexico) abbreviated version, *M* = 107, *SD* = 10) were recruited through personal invitation and social networks. All of them had an undergraduate degree or were studying at the time of the examination, with at least 1 year in their college program (years of schooling: *M* = 15, *SD* = 1). We estimated each individual’s working memory index using the WAIS-IV’s arithmetic and digit span tests (*M* = 99, *SD* = 9). An interview, brief clinical history, and the WAIS-IV tests were applied to the participants before the EEG recording and the experimental task. Also, the participants were instructed not to ingest any caffeinated beverages (including tea) 24 h prior to the tests, and to sleep well the night before the experiment. As compensation for participating in the study, the participants received a brief report of their estimated IQ.

None of the participants had antecedent clinical neurodevelopmental, neurological, or psychiatric conditions, nor did they report memory- or attention-specific complaints during the interview. Ethical approval was received before testing from the Neuroscience Institute’s Ethics Committee (ET022023-359). All participants voluntarily agreed to participate and provided written informed consent.

### 2.2. Sternberg Item Recognition Task

**Figure 1:**
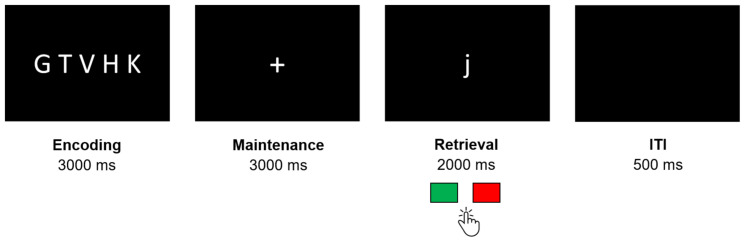
One trial of the modified Sternberg item recognition paradigm. The participants had to press the green button if the letter presented during retrieval was within the string of letters presented at encoding, otherwise they had to press the red button. ITI “inter-trial-interval”.

The experimental task was a modified Sternberg task with three levels of memory load. The stimuli consisted of strings of three (low memory load), five, and six capital consonant letters (higher memory load). All the letters constituting one string were presented in white on a black background, separated by a space from one another. Each string appeared at the center of a screen for 3000 ms (a period we called the encoding stage, although this process might occur during the first lapse of this time [4]) and was followed by a period of 3000 ms with only a central fixation point (maintenance stage). Later, the maintenance period was followed by the appearance of a centrally located white lowercase letter lasting 2000 ms (retrieval stage) in which the individual had to respond by pressing, as fast as possible, a green button if the letter corresponded to its capital one in the preceding string or a red button if it did not. A 500 ms inter-trial interval followed this period, and the subsequent trial began immediately afterward. The position of the target digit within the memorized sequence (e.g., first digit of the sequence, second digit of the sequence, etc.) was completely randomized. The schematic experimental flow is shown in Figure 1.

A total of 120 trials (40 for each memory load condition) were administered. The stimuli were presented in 4 blocks of 30 pseudo-randomized trials (10 for each memory load level). All trials within a block were presented automatically, one after the other, until the end of the block. The participants had a brief break between blocks, starting the next block at their own pace. A direct visual-matching strategy was prevented using lowercase letters as probes.

Before the experimental session, participants had a practice block of seven trials, consisting of numbers for the strings and probes, while maintaining the same temporal parameters.

We used Compumedics Neuroscan (Charlotte, NC, USA) Stim2 software (4.3.2) to program and run the task, and a Cedrus’ StimTracker Duo interface and RB-740 response pad (both devices from Cedrus Corporation, San Pedro, CA, USA) to record behavioral responses. Participants were seated in a dimly lit and sound-attenuated room, ~60 cm distance from a 27-inch AOC 27G2 monitor (AOC International, Amsterdam, Netherlands) with a 60 Hz refresh rate and 1024×768×32 screen resolution set in the software settings. The font style was Arial, with a point size of 29; this corresponded to a height of ~1.9 cm per stimulus. The visual angle was ~1.81°.

### 2.3. EEG Recording and Processing

EEG data were acquired using a Compumedics Neuroscan Grael 4K EEG amplifier (Charlotte, NC, USA), a 32-channel Quick-Cap Neo Net (Compumedics Neuroscan, Charlotte, NC, USA), and Curry 9 X software (9.0.2). EEG was recorded from 28 channels according to the 10/20 system (Fp1, Fp2, F7, F3, Fz, F4, F8, FC3, FCz, FC4, T7, C3, Cz, C4, T8, CP3, CPz, CP4, P7, P3, Pz, P4, P8, O1, Oz, O2, M1, M2), with four additional periocular bipolar channels to record blinks and eye movements. The reference electrode during the recording was located between Cz and CPz. The sampling rate was 2048 Hz, and the interelectrode impedance was below 5 kΩ.

The continuous EEG data were first preprocessed and segmented into non-overlapping epochs using EEGLAB v2024.0 [30] functions and custom MATLAB (R2023b) scripts. The data were downsampled to 500 Hz and filtered with a 0.05 Hz high-pass finite-impulse response (FIR) filter (filter order 33001, transition bandwidth 0.05 Hz, cutoff 0.025 Hz). Line noise (60 Hz) was removed using the CleanLine plugin with the default options, and the Clean Rawdata plugin (v2.11) was used only to identify flatline and noisy channels, also with the default options (no Artifact Subspace Reconstruction, and no bad data periods were removed using this plugin). A spherical spline method was used to interpolate noisy channels. Afterward, the recording was referenced to linked mastoids. To optimize artifact separation, independent component analysis (ICA) was estimated on a copy of the data that had been high-pass filtered at 1 Hz (filter order 1651, transition bandwidth 1 Hz, cutoff 0.05 Hz), which reduces the influence of slow drifts and improves component decomposition [31]. The ICA decomposition was performed using the pop_runica function, which estimates the effective data rank and performs PCA-based dimensionality reduction when rank-deficient data are identified. The resulting ICA weights were then applied unchanged to the minimally filtered data (0.05 Hz high-pass) to preserve low-frequency neural activity for subsequent analysis. Visual inspection was conducted by an independent neurophysiologist according to predefined criteria. Epochs containing residual eye movements, muscle artifacts, amplifier saturation, or abrupt non-physiological transients that were not adequately removed by ICA were rejected based on their spectral and temporal EEG characteristics. Trials associated with incorrect responses were also excluded from further analysis. Finally, the data was re-referenced to the common averaged reference for subsequent analysis.

After preprocessing, the first second of each processing stage was selected for analysis with the aim of maximizing comparability across task stages while reducing overlap between adjacent cognitive operations and minimizing contamination from late response-related activity. For each subject, we kept the same number of epochs across conditions (memory load and processing stage) with a group mean of 23 epochs (*SD* = 3). The subject with the least number had 20 epochs, and the subject with the maximum had 32 epochs (20 and 8 trials rejected, respectively). The power spectrum was calculated using Fast Fourier Transform (FFT) with a Hamming window of 500 data points and a 250-point overlap. Finally, we analyzed the mean absolute power in each frequency band (theta: 4–7 Hz, lower alpha: 8–10 Hz, upper alpha: 11–13 Hz) for each processing stage and memory load condition. Absolute power was also computed for the inter-trial interval and used as a baseline reference. Task-related power was normalized relative to this baseline using a decibel (dB) transformation.

### 2.4. Analysis

We used a repeated measures analysis of variance (ANOVA) to identify differences in accuracy (percentage of correct responses) and reaction time (milliseconds) between the different levels of memory load. The Greenhouse–Geisser correction was applied when necessary, and a Bonferroni correction was used for multiple comparisons.

Electrophysiological data were analyzed using cluster-based permutation testing for each contrast, an approach that leverages the spatial dependency structure of EEG signals. Rather than evaluating each electrode independently, this method tests whether there are spatially contiguous clusters of electrodes showing consistent effects that are unlikely to arise by chance. At each iteration, a *t*-test was computed at every electrode, and values exceeding the critical threshold of *t*(44) = 2.01 (corresponding to *p* < 0.05, uncorrected) were retained. Spatially adjacent significant electrodes were grouped into clusters, and the *t*-values within each cluster were summed to obtain the cluster-level statistic (*t*-sum). The maximum cluster-level statistic from each iteration was used to construct the null distribution. This procedure was repeated 5000 times. Clusters were defined based on a maximum inter-electrode distance of 40 mm.

To control for multiple comparisons across contrasts, a global null distribution of maximum cluster-level statistics was generated by selecting the highest cluster-level value across all contrasts at each permutation. Cluster-level statistics derived from the observed data were then compared against this global null distribution to determine statistical significance. This procedure was conducted separately for each frequency band. For significant clusters, effect sizes were estimated using Cohen’s *d*, calculated using the mean absolute power of the elements comprising a cluster.

As an additional exploratory analysis of the relationship between absolute power magnitude and behavioral performance, generalized linear models were fitted. Accuracy was analyzed using a binomial generalized linear model with absolute power as the predictor and number of correct responses as the dependent variable. Reaction time was analyzed using linear regression models. Each frequency band was tested separately. Because analyses were conducted for each electrode and condition, false discovery rate (FDR) correction was applied to control for multiple comparisons. Only electrodes belonging to significant clusters were included in these regression analyses.

## 3. Results

### 3.1. Behavioral Data

The ANOVA showed a significant effect of memory load on accuracy (*F*(2, 88) = 60.7, *p* < 0.001, *η*^2^ = 0.58), with accuracy decreasing as memory load increased. The percentage of correct responses was higher at the three-letter memory load (*M* = 95.44, *SD* = 3.93) than at the five letter (*M* = 87.22, *SD* = 7.69, *p* < 0.001) or six letter (*M* = 85, *SD* = 6.87, *p* < 0.001) memory loads. There was no significant difference between five and six letters.

The memory load also had a significant effect on reaction time, which increased with the increase in memory load (*F*(1.72, 75.76) = 61.8, *p* < 0.001, *η*^2^ = 0.58). Reaction time was higher in the memory load of five letters (*M* = 921.64, *SD* = 115.68, *p* < 0.001), and six letters (*M* = 926.26, *SD* = 148.68, *p* < 0.001) compared to three letters (*M* = 816.58, *SD* = 111.03). There was no significant difference in reaction time between five and six letters.

### 3.2. Electrophysiological Data

#### 3.2.1. Theta

*Processing-stage effects:* Theta power was significantly higher during retrieval than during encoding across memory loads. At three letters, this effect involved bilateral and midline fronto-central-parietal regions (*p* < 0.01). At five letters, the effect was restricted to left and midline fronto-central-parietal regions (*p* < 0.05). At six letters, it was limited to left fronto-central regions (*p* < 0.05).

**Figure 2:**
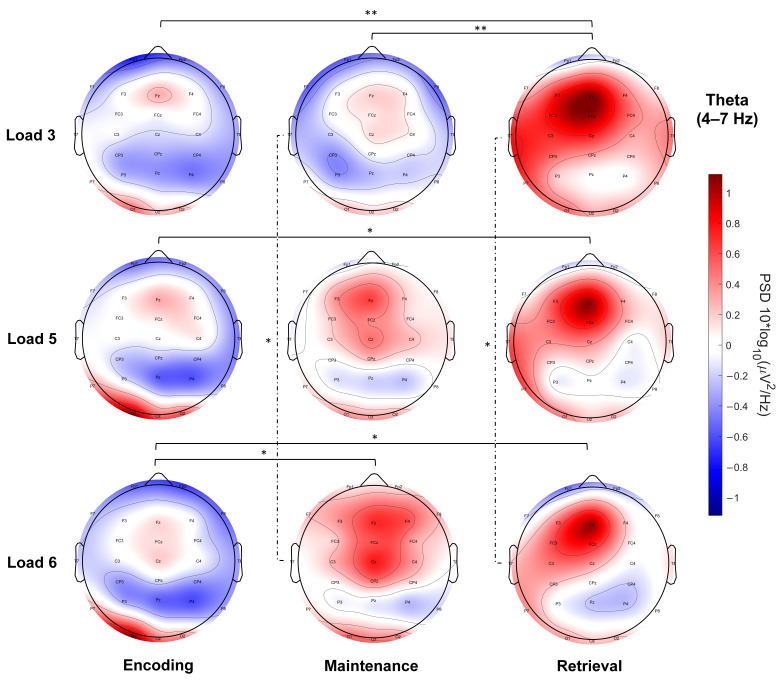
Absolute power of the theta frequency band in each condition and working memory processing stage averaged across participants. The figure shows a significantly higher theta power, primarily distributed across frontal regions, during retrieval across memory loads. The specific cluster statistic values and *p* values are in Table 1. * *p* < 0.05; ** *p* < 0.01.

Additionally, at three letters, retrieval elicited higher theta power than maintenance in left and midline regions (*p* < 0.01). At six letters, maintenance showed higher theta power than encoding in right and midline regions (*p* < 0.05). Figure 2 shows the absolute power of the theta frequency band per condition and working memory processing stage, while Table 1 summarizes the results.

*Memory load effects*: During maintenance, theta power was higher at six letters compared to three letters in a midline fronto-central cluster (*p* = 0.035). In contrast, during retrieval, theta power was higher at three letters compared to six letters in right fronto-central regions (*p* = 0.049).

*Brain–behavior association*: In the three-letter condition, increased theta power at FCz during retrieval predicted faster responses, *b* = −43.00, *SE* = 10.78, *t*(43) = −3.99, *p* < 0.001, *r*^2^ = 0.27. No associations with accuracy were observed.

Overall, theta activity was primarily enhanced during retrieval and was behaviorally relevant under low memory load.

#### 3.2.2. Lower Alpha

*Processing-stage effects*: Stage-related differences varied by memory load. At three letters, maintenance elicited higher lower-alpha power than encoding in midline fronto-central-parietal regions (*p* = 0.006). Maintenance also exceeded retrieval in bilateral fronto-central and parietal regions (*p* ≤ 0.001).

At five and six letters, encoding elicited higher lower-alpha power than retrieval (left hemisphere at five letters, *p* < 0.001; bilateral at six letters; all *p* < 0.05). Also, at both memory load levels, maintenance exceeded retrieval in bilateral and midline fronto-central-parietal regions (all *p* ≤ 0.008).

*Memory load effects:* During maintenance, lower alpha power was higher at three letters than at six letters in occipital regions (*p* = 0.022). No significant associations were found between lower alpha power and behavioral performance.

**Figure 3:**
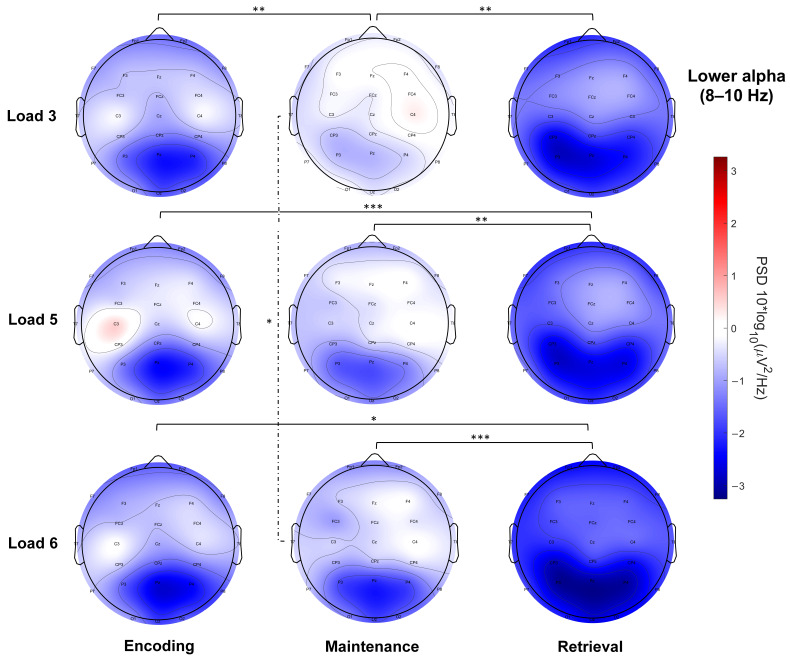
The absolute power of the lower alpha frequency band in each condition and working memory processing stage averaged across participants. The figure shows how lower alpha power decreases with higher memory load, especially during maintenance. The specific cluster statistic values and *p* values are in Table 2. * *p* < 0.05; ** *p* < 0.01; *** *p* < 0.001.

In summary, lower alpha power was predominantly elevated during maintenance, particularly under lower load, whereas encoding-related increases emerged at higher loads. Figure 3 shows the absolute power of the lower alpha frequency band for each condition and working memory processing stage, while Table 2 summarizes the results.

#### 3.2.3. Upper Alpha

Upper alpha showed the most consistent and robust effects across conditions.

*Processing-stage effects:* Across all memory loads, maintenance elicited higher upper alpha power than encoding in midline and right fronto-central-parietal regions, as well as occipital areas (all *p* ≤ 0.005). Also, maintenance exceeded retrieval in bilateral fronto-central-parietal and occipital regions (all *p* < 0.001), with large effect sizes (many *d* > 1). Finally, encoding elicited higher upper alpha power than retrieval in left fronto-central-parietal regions across all loads (all *p* < 0.001). Thus, upper alpha activity was maximal during maintenance, regardless of memory load.

*Memory load effects:* During retrieval, upper alpha power was lower at six letters compared to three letters in midline fronto-central-parietal regions (*p* = 0.006).

**Figure 4:**
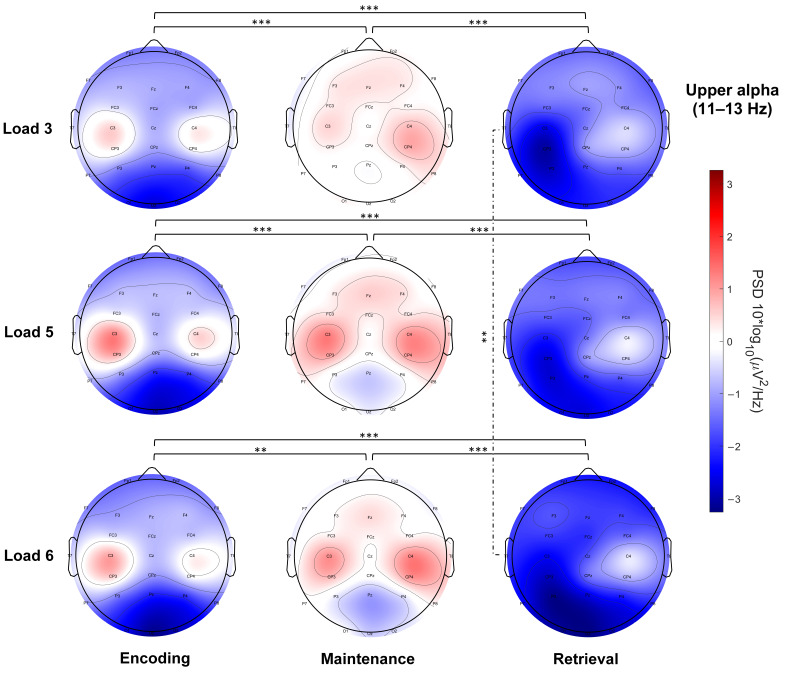
The absolute power of the upper alpha frequency band in each condition and working memory processing stage averaged across participants. The figure shows that upper alpha power is significantly higher during maintenance, regardless of the memory load. The specific cluster statistic values and *p* values are in Table 2. ** *p* < 0.01; *** *p* < 0.001.

*Brain–behavior associations:* In the five-letter condition, higher upper alpha power was associated with a greater probability of correct responses across encoding, maintenance, and retrieval stages in left fronto-central and occipital regions (FDR-corrected *p* ≤ 0.044). Odds ratios ranged from 1.08 to 1.18, indicating that increased upper alpha power significantly improved the likelihood of correct responses. No associations were observed between upper alpha power and reaction time. Figure 4 shows the absolute power in the upper alpha frequency band for each condition and working memory processing stage, while Table 3 summarizes statistical analysis results.

## 4. Discussion

The present study examined how theta, lower alpha, and upper alpha oscillations are modulated across temporally dissociated stages of working memory (WM) processing using a modified Sternberg paradigm. By separating encoding, maintenance, and retrieval processes, the study addressed an important limitation of paradigms such as the n-back task, in which multiple cognitive operations overlap temporally, complicating stage-specific interpretations [28,32]. Overall, the findings revealed a dissociation between theta and alpha oscillatory dynamics across WM stages. Theta activity was predominantly enhanced during retrieval and showed selective associations with response speed, whereas alpha activity, particularly upper alpha, was maximal during maintenance and was associated with behavioral accuracy under moderate memory load. These findings suggest that distinct oscillatory processes contribute differently to WM operations across task stages and cognitive demands.

The modified Sternberg task also introduced additional executive requirements during retrieval because probes were presented as lowercase letters, whereas the memory set consisted of uppercase letters. Consequently, participants likely had to perform a case-transformation operation before comparing the probe to the maintained representations. This manipulation was intended to reduce reliance on direct perceptual matching strategies, but it also increased executive and decision-related processing demands during retrieval. Behavioral performance confirmed that the memory-load manipulation was effective, as higher loads produced lower accuracy and slower reaction times, consistent with capacity-limited models of WM and cognitive load theory [2,5,6,7].

### 4.1. Theta Oscillations and Retrieval-Related Processing

Theta oscillations showed the clearest enhancement during retrieval across memory loads, particularly over fronto-central regions. This pattern is consistent with the previous literature linking frontal–midline theta activity to executive control, attentional allocation, conflict monitoring, and WM performance [14,15,33,34,35,36,37]. However, the present findings extend previous work by demonstrating that when WM stages are temporally dissociated, theta activity is more strongly associated with retrieval-related operations than with encoding or maintenance alone.

Importantly, the present data should not be interpreted as direct mechanistic evidence that theta selectively indexes mnemonic retrieval. Reasonably, the findings suggest that retrieval-stage theta may reflect the coordinated engagement of several cognitive operations required for memory-guided responding, including retrieval, comparison processes, executive coordination, response selection, and motor preparation. This interpretation is particularly relevant because EEG epochs during retrieval were stimulus-locked rather than response-locked, meaning that motor-related processes almost certainly contributed to the observed activity. The fronto-central topography of the theta enhancement is compatible not only with executive control processes but also with premotor and response-preparation mechanisms [33,38,39]. Therefore, the retrieval-related theta increase observed here is more appropriately interpreted as probably reflecting integrated cognitive-control and response-related processes during memory-guided decisions.

The behavioral relevance of theta oscillations was supported by the finding that greater theta power during retrieval was associated with faster reaction times under low memory load. Although this analysis was exploratory, the association suggests that increased theta synchronization may facilitate efficient coordination between retrieval and response-selection processes when cognitive demands remain manageable. Similar associations between frontal theta synchronization and efficient cognitive performance have been reported previously in WM and attentional-control paradigms [15,16,36].

Theta activity also exhibited load-dependent modulation, although these effects were less robust and more spatially restricted than the stage-related differences. During maintenance, theta power increased under higher-load conditions, particularly in midline fronto-central regions. This finding agrees with studies reporting load-dependent increases in frontal theta activity during WM tasks [40,41,42]. However, the present results additionally indicate that retrieval-related theta enhancement remained dominant even under higher load conditions. Together, these findings suggest that theta oscillations may contribute to both sustained cognitive control during maintenance and retrieval-related executive coordination, with their relative contribution depending on task stage and processing demands.

Although hippocampal–cortical interactions have frequently been proposed as generators of WM-related theta oscillations [43,44,45], the present scalp EEG data do not permit direct inference regarding deep neural sources. Consequently, the observed effects are more prudently interpreted as reflecting large-scale fronto-parietal coordination associated with retrieval and executive processing rather than direct evidence of hippocampal activity.

### 4.2. Alpha Oscillations and Maintenance-Related Stabilization

In contrast to theta activity, alpha oscillations demonstrated highly consistent stage-related modulation across all memory loads. Both lower and upper alpha power were maximal during maintenance compared with encoding and retrieval, suggesting that alpha activity may contribute preferentially to processes involved in maintaining and stabilizing information over time.

Traditionally, increases in alpha power during WM maintenance have been interpreted within an inhibitory-gating framework, according to which alpha synchronization suppresses irrelevant sensory input to protect internally maintained representations [14,16,46,,47]. The present findings are broadly consistent with this interpretation because maintenance-related alpha increases were observed across widespread fronto-central, parietal, and occipital regions. Such increases may reflect top-down mechanisms that reduce interference from external stimulation during active retention.

However, inhibitory gating is not the only possible interpretation of maintenance-related alpha synchronization. Alternative accounts propose that alpha oscillations may support internally directed attention, predictive timing, perceptual disengagement, or top-down stabilization of mnemonic representations [12,47,48]. The present findings cannot distinguish conclusively among these explanations. Nevertheless, the robust enhancement of alpha activity during maintenance supports the broader interpretation that alpha oscillations help preserve task-relevant internal representations when external sensory input is minimal.

Lower and upper alpha bands also exhibited partially distinct modulation patterns, supporting proposals that these sub-bands serve different functional roles [14,16,49,50,51]. Lower alpha activity showed some sensitivity to memory load, particularly in occipital regions during maintenance, where alpha power decreased under higher load conditions. This reduction may indicate diminished sensory inhibition or increased recruitment of posterior cortical resources as mnemonic demands increase [52]. In contrast, upper alpha activity exhibited the strongest and most widespread maintenance-related enhancement across all load conditions.

Notably, upper alpha power predicted a greater probability of correct responses in the five-letter condition across the encoding, maintenance, and retrieval stages. Although these analyses were exploratory and should be interpreted cautiously, they suggest that stronger upper-alpha synchronization may support successful performance when task demands approach WM capacity limits. Previous studies have associated upper alpha oscillations with semantic processing, cognitive control, and active maintenance processes [14,16,49,50,51], and the present results are consistent with the possibility that upper alpha contributes to the stabilization and efficient manipulation of maintained representations during demanding cognitive operations.

Interestingly, alpha oscillations did not show a robust global load effect across conditions. This finding differs somewhat from studies reporting monotonic increases in alpha with increasing WM load [12,16]. One possible explanation is that the present task emphasized stage-specific processing rather than continuous updating, as is common in n-back paradigms. Consequently, maintenance-related alpha synchronization may primarily reflect qualitative differences in attentional stabilization processes rather than a simple quantitative increase in cognitive demand.

### 4.3. Functional Dissociation Between Theta and Alpha Oscillations

The principal contribution of the present study is the demonstration of a stage-dependent dissociation between theta and alpha oscillations during WM processing. Theta activity was predominantly retrieval-related and associated with response speed, whereas alpha activity was maintenance-dominant and associated with successful task performance under moderate load. These findings suggest that theta and alpha oscillations may contribute to complementary but partially independent aspects of WM processing.

More specifically, theta oscillations may reflect processes involved in executive coordination, retrieval, decision-making, and response preparation, whereas alpha oscillations may support the stabilization of internally maintained representations through inhibitory or attentional-control mechanisms. Importantly, the present results do not establish direct causal or mechanistic relationships between these oscillations and specific cognitive operations. Rather, the findings indicate that stage-specific oscillatory dynamics are compatible with current neurocognitive models proposing differentiated functional roles for theta and alpha activity during WM [14,15,16].

The use of a temporally dissociated Sternberg paradigm was particularly important for clarifying these dynamics. In many previous WM studies, especially those using n-back paradigms, encoding, updating, maintenance, and retrieval occur simultaneously, making it difficult to determine whether oscillatory changes reflect a single process or multiple overlapping operations [28,32]. By separating these stages, the present design provided clearer evidence that theta and alpha oscillations are differentially modulated depending on the temporal demands of the task.

Interestingly, our initial hypothesis predicted a progressive decrease in alpha power with increasing task engagement. Instead, alpha activity increased robustly during maintenance. This finding supports contemporary perspectives suggesting that alpha synchronization may reflect an active regulatory mechanism that protects internal representations rather than passive cortical idling [27,47,48]. Thus, the present study contributes to a growing body of evidence indicating that increased alpha power during maintenance reflects functional engagement rather than reduced neural processing.

### 4.4. Limitations and Future Directions

Several limitations should be considered when interpreting the present findings. First, the relatively short inter-trial interval may have allowed residual post-response activity to influence the baseline period used for normalization. Although the same baseline procedure was applied consistently across all conditions, residual motor- or decision-related activity may have contributed particularly to theta-band estimates. Consequently, baseline-normalized power differences should be interpreted cautiously.

Second, retrieval-related activity likely included motor preparation and response selection processes, as retrieval epochs were stimulus-locked and temporally overlapped with behavioral responses. Although the first second of each stage was selected to minimize contamination from late-response activity and maximize comparability across stages, the retrieval window almost certainly included non-mnemonic cognitive processes. Future studies using response-locked analyses or shorter pre-response windows may help isolate retrieval-specific oscillatory activity more precisely.

Third, FFT-based absolute power estimation provides limited temporal resolution and therefore cannot fully characterize dynamic fluctuations in oscillatory activity within each processing stage. Time–frequency approaches such as wavelet convolution or short-time Fourier transform analyses may provide a more detailed characterization of evolving oscillatory dynamics during WM processing.

Additional methodological considerations should also be acknowledged. Fixed alpha sub-band boundaries were used to facilitate comparability with the previous WM literature, although individual alpha frequency approaches may provide greater physiological specificity. Furthermore, the exploratory brain–behavior analyses were conducted using electrodes identified through cluster-based analyses, which may increase the risk of circular inference [53,54,55]. Consequently, these associations should be interpreted cautiously until replicated using independent region-of-interest approaches or cross-validation procedures.

Finally, although incorrect trials were excluded to ensure greater signal quality and interpretability, this prevented direct comparison between successful and unsuccessful memory performance. Future studies incorporating more error trials, individual performance profiles, and time–frequency analyses may further clarify the oscillatory mechanisms underlying WM success and failure.

## Figures and Tables

**Table 1:** Summary of the statistical results for the effects of processing stage and memory load on the theta band absolute power.

Comparison	Memory Load	Direction of Effect	Main Topography	*t*-Sum	*p*	*d*
Encoding vs. Retrieval	3 letters	Encoding < Retrieval	Bilateral and midline fronto-central-parietal	−21.32–−14.54	<0.001–0.008	0.55–0.75
	5 letters		Left and midline fronto-central-parietal	−17.24–−12.31	0.001–0.036	0.53–0.71
	6 letters		Left fronto-central	−14.02	0.013	0.66
Maintenance vs. Retrieval	3 letters	Maintenance < Retrieval	Left and midline fronto-central-parietal	−22.84–−16.51	<0.001–0.002	0.67–0.69
Encoding vs. Maintenance	6 letters	Encoding < Maintenance	Right and midline fronto-central-parietal	−14.49–−13.09	0.009–0.024	0.58–0.60
Memory load effect (Maintenance)	3 < 6 letters	Higher theta at 6 letters	Midline fronto-central	−12.38	0.035	0.56
Memory load effect (Retrieval)	3 > 6 letters	Higher theta at 3 letters	Right fronto-central	11.36	0.049	0.38
Brain–behavior association	3 letters (Retrieval)	Higher theta-lower reaction time	FCz (fronto-central)		<0.001	*r*^2^ = 0.27

Note. Topography summarizes significant electrode clusters. The values are the minimum and maximum values of each statistic (*t*-sum, *p*, and Cohen’s *d*). In the brain–behavior association, regression results indicated that higher theta power was associated with faster reaction times.

**Table 2:** Summary of the statistical results for the effects of processing stage and memory load on the lower alpha band absolute power.

Comparison	Memory Load	Direction of Effect	Main Topography	*t*-Sum	*p*	*d*
Encoding vs. Maintenance	3 letters	Encoding < Maintenance	Midline fronto-central-parietal	−16.7	0.006	0.54
Encoding vs. Retrieval	5 letters	Encoding > Retrieval	Left fronto-central-parietal	23.2	<0.001	0.85
	6 letters		Bilateral fronto-central-parietal	14.7–20.4	0.03–<0.001	0.59–0.76
Maintenance vs. Retrieval	3 letters	Maintenance > Retrieval	Bilateral and midline fronto-central-parietal	19.1–27.8	≤0.001	0.65–0.95
	5 letters			16.5–20.6	0.01	0.47–0.67
	6 letters			24.3–27.1	<0.001	0.56–0.72
Memory load effect (Maintenance)	3 > 6 letters	Higher lower alpha at 3 letters	Occipital	15.0	0.02	0.63

Note. Topography summarizes significant electrode clusters. The values are the minimum and maximum values of each statistic (*t*-sum, *p*, and Cohen’s *d*).

**Table 3:** Summary of the statistical results for the effects of processing stage and memory load on the upper alpha band absolute power.

Comparison	Memory Load	Direction of Effect	Main Topography	*t*-Sum	*p*	*d*
Encoding vs. Maintenance	3 letters	Encoding < Maintenance	Midline fronto-central-parietal; occipital	−23.31–−23.28	<0.001	0.82–1.23
	5 letters		Midline and right fronto-central-parietal; occipital	−23.31–−20.59	<0.001	0.65–1.11
	6 letters			−18.94–−17.45	0.002–0.005	0.60–0.98
Maintenance vs. Retrieval	3 letters	Maintenance > Retrieval	Bilateral and midline fronto-central-parietal; occipital	22.42–41.23	<0.001	0.95–1.54
	5 letters			22.41–43.96	<0.001	0.94–1.52
	6 letters			24.77–48.19	<0.001	0.92–1.38
Encoding vs. Retrieval	3 letters	Encoding > Retrieval	Left fronto-central-parietal	26.06	<0.001	1.39
	5 letters			29.66	<0.001	1.16
	6 letters			29.43	<0.001	1.16
Memory load effect (Retrieval)	3 > 6 letters	Higher upper alpha at 3 letters	Midline fronto-central-parietal	17.07	0.006	0.38
Brain–behavior association	5 letters	Higher upper alpha higher accuracy	Left fronto-central and occipital		≤0.044 (FDR)	OR = 1.08–1.18

Note. Topography summarizes significant electrode clusters. The values are the minimum and maximum values of each statistic (*t*-sum, *p*, and Cohen’s *d*). In the brain–behavior association analysis, odds ratios (OR) reflect increased probability of correct responses associated with higher alpha power (false discovery rate-corrected *p*-values).

## Data Availability

The data presented in this study are available on request from the corresponding author due to privacy restrictions.
